# ¿Esperamos demasiado de los comités de ética en investigación existentes?

**DOI:** 10.18294/sc.2025.5782

**Published:** 2025-07-11

**Authors:** Núria Homedes, Antonio Ugalde

**Affiliations:** 1 Médica. Doctora en Salud Pública. Ex profesora y directora, Global Health, School of Public Health, University of Texas Health Science Center at Houston, EEUU. nhomedes@gmail.com University of Texas Health Science Center at Houston School of Public Health University of Texas Health Science Center at Houston USA nhomedes@gmail.com; 2 Doctor en Derecho. Doctor en Sociología. Profesor emérito, Department of Sociology, University of Texas at Austin, EEUU. augalde@utexas.edu University of Texas at Austin Department of Sociology University of Texas at Austin USA augalde@utexas.edu

## LOS ORÍGENES

La necesidad de establecer criterios éticos para investigar en seres humanos surgió a raíz de los juicios de Nuremberg, cuando se conocieron las atrocidades cometidas durante los experimentos biomédicos de la Segunda Guerra Mundial. El código de Nuremberg de 1947^1^ ha tenido mucha influencia en las pautas éticas de la investigación en humanos que los países han ido elaborando e incorporando en sus marcos regulatorios. 

En EEUU, en 1953, los *National Institutes of Health* empezaron a exigir que todos los proyectos de investigación clínica intramuros obtuvieran la aprobación de un panel de revisión; y, en 1966, el *US Public Health Service* extendió este requisito a los protocolos de investigación extramuros que financiaba la agencia. En la década de 1970 se fueron estableciendo comités de ética en investigación (CEI) -que en EEUU se conocen con el nombre de *institutional review boards* (IRBs)- en cientos de instituciones que recibían financiación federal^2^ y, en 1974, el *US Department of Health and Human Services* emitió la primera regulación para proteger a los sujetos humanos^3^, e incluyó el requisito de la revisión ética grupal^4^. En 1975, la Asociación Médica Mundial incorporó la revisión de los protocolos de investigación por parte de un CEI en la Declaración de Helsinki^5^. 

El 1981, la *National Commission for the Protection of Human Subjects of Biomedical and Behavioral Research* de EEUU elaboró el Informe Belmont, en el que se exponen los principios éticos subyacentes a la conducta relacionada con la investigación en humanos^6^. Estos principios, que incluyen la revisión de los protocolos por parte de un CEI, se incorporaron en las regulaciones del *US Department of Health and Human Services* de 1981, y en 1991 otras 16 agencias federales adoptaron la *Common Rule*^4^, ampliando su ámbito de aplicación más allá de la investigación biomédica. 

Con el tiempo, la mayoría de los países -incluyendo los de América Latina- han ido desarrollado sus propias leyes y regulaciones para controlar la investigación que involucra a seres humanos, siguiendo el modelo de EEUU^7^. La mayoría de estos países han otorgado a los CEI la responsabilidad de proteger a los participantes en investigación y la capacidad de decidir, entre otras cosas, si un protocolo de investigación que involucra a seres humanos puede ser implementado o no^8^. 

## PROTOCOLOS SOCIALES

El problema es que los requisitos establecidos para proteger a los participantes en experimentos biomédicos, en los que se utilizan medicamentos o dispositivos experimentales, se utilizan para evaluar todos los protocolos que requieren que el investigador interactúe con seres humanos, incluyendo estudios de políticas públicas, organizaciones sociales y estudios antropológicos (de ahora en adelante nos referiremos a ellos como “protocolos sociales”). Unos pocos países han establecido normas específicas para evaluar los protocolos sociales, y un grupo internacional de académicos emitió la *New Brunswick Declaration*, pero su impacto ha sido limitado^9^. 

La normativa suele exigir que estos CEI cuenten con al menos cinco miembros, de diferentes disciplinas, entre los que debe haber alguien que sepa de ética, algún experto en métodos de investigación, alguien que represente a los no científicos para que provean la opinión de la comunidad afectada y verifiquen que no se violen sus derechos y, a veces, un abogado. Una vez constituido, el CEI suele revisar todo tipo de proyectos, desde ensayos clínicos financiados por la industria farmacéutica y de dispositivos médicos, hasta etnografías y trabajos de final de curso o final de carrera^10^^,^^11^^,^^12^^,^^13^^,^^14^^,^^15^. 

Es fácil entender que las entrevistas, observaciones etnográficas, grupos focales, e incluso las intervenciones conductuales conllevan menores riesgos que las intervenciones biomédicas^16^. Sin embargo, al hacer su evaluación, los CEI utilizan el paradigma biomédico, sin tener en cuenta que los riesgos para los participantes en un protocolo social suelen ser mínimos y muy distintos. Es más, los programas de formación en ética que deben tomar los miembros de los CEI, están basados en los riesgos de la investigación biomédica^10^^,^^11^^,^^12^^,^^13^^,^^14^^,^^15^.

Algunos científicos sociales se quejan de que los criterios y el lenguaje que exigen los CEI no se adecua a sus disciplinas, de que los CEI les bloquean protocolos de riesgo mínimo que hubieran aportado información importante, y de que los CEI sobreprotegen a las comunidades/individuos, negándoles la capacidad de decidir por sí mismos si quieren o no conceder una entrevista o participar en una intervención conductual^17^^,^^18^. 

No cuestionamos que haya casos en que la investigación observacional y cualitativa puede violar los derechos de poblaciones vulnerabilizadas, estigmatizar a ciertos grupos, ser demasiado intrusiva, e incluso inducir conductas inapropiadas. Por ejemplo, ciertas categorías de participantes podrían experimentar riesgos si no se toman medidas especiales para proteger su identidad, por ejemplo, los inmigrantes irregulares o la población indocumentada, las personas con padecimientos mentales, los usuarios de drogas o ciertas minorías étnicas. Algunos experimentos para controlar enfermedades transmitidas por insectos podrían tener repercusiones negativas para el ecosistema, y podrían conllevar más riesgos que beneficios para comunidades muy amplias. Estudios sobre conductas sexuales y adicciones pueden poner en riesgo a los participantes, del mismo modo que los estudios con muestras pequeñas pueden dificultar que se mantenga la confidencialidad de los informantes. Además, existen protocolos sociales que carecen de valor social y/o científico y no se considerarían éticos.

Quizás uno de los elementos que dificulta la evaluación de dichos protocolos, es que los riesgos de participar en una investigación social o cualitativa no están bien definidos y, consecuentemente, ni los marcos regulatorios ni los programas de formación de miembros de CEI los abordan adecuadamente. Además, como los CEI suelen estar ubicados en establecimientos que también hacen investigación biomédica, es frecuente que el metodólogo que participa en el CEI no sepa mucho sobre la investigación cualitativa, y se sienta incómodo con su carácter abierto. Por otra parte, el formato para registrar la evaluación que han hecho los CEI no encaja con la información que se incluye en los protocolos sociales, y mientras no se espera que un antropólogo evalúe si el participante en un ensayo clínico se expone a riesgos innecesarios, sí se suele permitir que un matemático o alguien con experiencia en ensayos clínicos evalúe estudios etnográficos o encuestas a la comunidad. 

Es decir, en el contexto actual, los científicos sociales están en desventaja cuando presentan sus proyectos al CEI, y se quejan de que los CEI no captan el valor social de sus investigaciones y, a veces, hacen críticas y exigen cambios a la metodología que les impiden responder a sus preguntas de investigación con el rigor que exige su disciplina^17^. 

Desde nuestra perspectiva, es irresponsable esperar que un CEI de cinco personas -o incluso siendo el doble de personas- pueda evaluar adecuadamente todo tipo de proyectos en investigación. Pensamos que lo primero que habría que hacer es profesionalizar a los CEI, y eso implica separar a los CEI que revisan proyectos biomédicos de los que revisan protocolos sociales. Los CEI que se dediquen a los protocolos sociales deberían empezar por reflexionar sobre los riesgos que enfrentan los seres humanos que participan en diversos tipos de investigación social, lo que permitiría establecer categorías de protocolos que estarían exentos de revisión por el CEI. 

Es posible que, al profesionalizar a los CEI y reducir el número de protocolos sociales que requieren revisión ética, se reduzca el número necesario de CEI para evaluar los protocolos sociales que puedan conllevar riesgos para los participantes. También se puede anticipar que los nuevos CEI no contarán con el conocimiento necesario para evaluar todo tipo de protocolos, sino que deberán tener acceso a expertos que puedan contribuir a dicha evaluación. Estos CEI “especializados” podrían estar ubicados en universidades y en entidades que financien los protocolos de investigación social, lo que les facilitaría el acceso a expertos en las diversas disciplinas involucradas.

El esfuerzo de los miembros de los CEI debe ser remunerado, o debe formar parte de la carga de trabajo que se les asigna. Proteger a los participantes en investigación es una tarea muy importante, que debe ser reconocida y remunerada.
